# Hemothorax after snake bite

**DOI:** 10.1002/ccr3.4874

**Published:** 2021-09-22

**Authors:** Mumen Abdalazim Dafallah, Elsanosi Habour, Esraa Ahmed Ragab

**Affiliations:** ^1^ Faculty of Medicine University of Gezira Wad Madani Sudan

**Keywords:** case report, hemothorax, snake bite, Sudan

## Abstract

Hemothorax after snake bite is very rare and unusual presentation. Administration of antivenom with supportive measures and close monitoring can prevent further deterioration. Delays in the transportation of patients to health facilities where antivenom and other therapeutic resources are provided can lead to devastating consequences.

## Graphical abstract

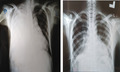

## 1 INTRODUCTION

Snake toxins are harmful and account for a significant degree of morbidity and mortality. Hematological abnormalities are the most important consequences of snake bite globally. We reported a case of snake bite in a Sudanese patient with the development of hemothorax as a complication. Snake bite is considered as an occupational crisis with high fatality rate especially in tropics.^1^ It affects mainly rural inhabitants and remote villages, particularly farmers and agricultural workers.^2^ The patient age, snake species, number and location of bites, and comorbid conditions affect the severity and the end result of snake bite victims.^3^ Renal failure, coagulopathy, bleeding, breathing problems, cardiac arrest, and compartment syndrome can be reported as complications.^4^, ^5^ We reported a case of snake bite with the development of hemothorax as a complication. To the best of our knowledge, this is the first case report of hemothorax after snake bite in Sudan.

## 2 CASE REPORT

A 21‐year‐old man from Sinnar, Central Sudan, referred to Wad‐Medani Teaching Hospital for the management of snake bite. He initially treated at Singa Teaching Hospital after snake bite to his left foot where he was admitted and received two units of blood, one unit of fresh frozen plasma and injectable antibiotics. The patient was referred to our hospital after 6 days without improvement for further workup and treatment.

On admission, there was localized abdominal pain at the umbilicus, hemoptysis, and hematuria associated with a decrease in urine amount and frequency, but there were no history of bleeding gums, epistaxis, hematemesis, and melena. There was no breathlessness, vomiting, headache, dizziness, focal neurological deficit, or paresthesia to the affected limb. The personal and past histories were insignificant.

On examination, he was oriented and conscious, not pale or jaundiced. His pulse rate was 112 beat/min, respiratory rate was 25 cycle/min, blood pressure was 100/70 mm of Hg, and his temperature was 39.3℃. The affected limb was swollen up to the knee with tenderness on palpation. There was ecchymosis around the affected area. Examination of the other systems was unremarkable.

The investigations were as follows: Complete blood count showed leukocytosis (14,000/mm^3^), and platelet count was 157/mm^3^ with low hemoglobin (8.6 g/dL). Urine, in general, showed uncountable RBCs and 2–4 pus cells. Blood urea was 44 mg/dL, serum creatinine was 0.5 mg/dL, serum sodium was 130 mEq/L, and serum potassium was 5.5 mmol/L. Random blood glucose was 88 mg/dL. The patient's prothrombin time (PT) was 20 seconds, activated partial thromboplastin time (aPTT) was prolonged 43 seconds, and the international normalization ratio (INR) was 1.6. On the first day of admission, the patient received one unit of blood, one unit of fresh‐frozen plasma every 6 h, antivenom, vitamin K, injectable antibiotics, and antipyretics.

**FIGURE 1:**
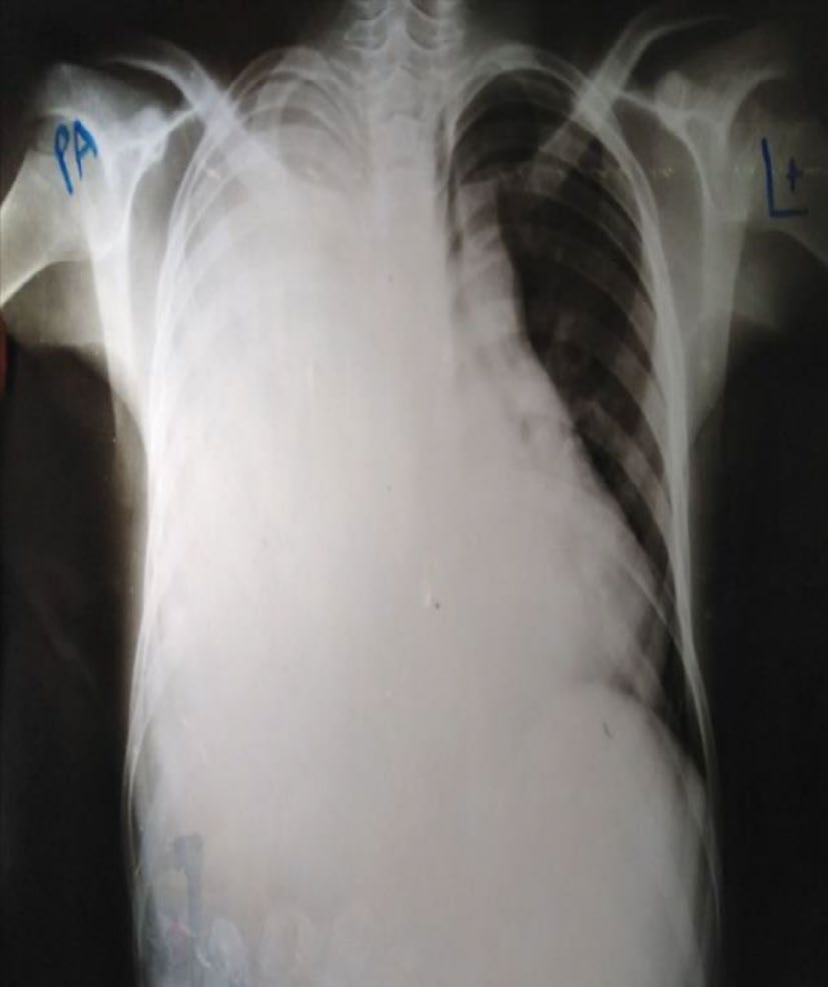
Poster‐anterior chest radiograph on admission (before the insertion of chest tube)

**FIGURE 2:**
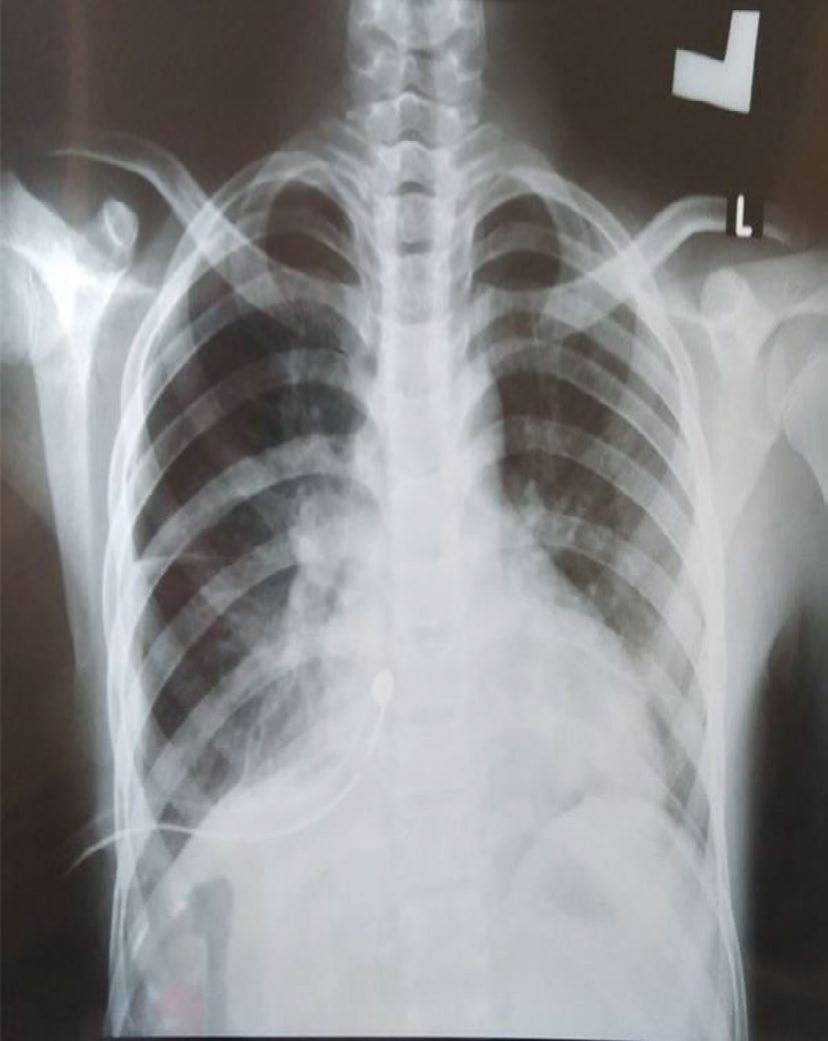
Poster‐anterior chest radiograph at discharge (after the insertion of chest tube**)**

On the fourth day of admission, the patient became pale and distressed. Chest examination showed that the right side of the chest moves less with percussive dullness and no air entry. Posterior‐anterior chest radiograph showed massive right‐side hemothorax. Immediately, 550 mL of blood was drained through needle inserted posteriorly in the second intercostal space midscapular line lateral to the spine and patient transfused with one unit of blood. Hours later, chest tube with under water seal was inserted and kept for further drainage and collection. On the following days, the tube drained about 3 L of blood. The patient's general condition improved gradually in the subsequent days and chest radiograph showed complete resolution of the hemothorax as shown in Figures 1 and 2. After which, chest tube with under water seal system was removed and the patient discharged in a good condition.

During hospitalization, the patient received six units of blood and thirty units of fresh‐frozen plasma, antivenom, vitamin K, injectable ceftriaxone, vancomycin, metronidazole, and antipyretics. On discharge, the patient's coagulation profile returns to normal limit.

## 3 DISCUSSION

Snake bite is a serious community health concern in sub‐Saharan Africa. More than 20,000 people die annually due to its harmful toxins.^6^ In Sudan, particularly the agricultural and rural areas have high incidence of snake bite; in fact, Sudan is considered a country with high death rate.^7^ Lack of community awareness of snake bite seriousness and in ability to seek medical advice and reaching the hospital in time makes it a life‐threatening emergency.^7^ A study from India reported that snake bite is more prevalent in children group, but there were limited data supporting this study.^8^ Unfortunately, the classification of snake species in Sudan has not been completed despite the discovery of more than 220 snake species.^9^ The Carpet Viper (*Echis carinatus*), the Black Burrowing Viper (*Atractaspis microlepidotus*), the Demon Night Adder (*Causus rhombeatus*), and the Lined House Snake (*Boodon lineatus*) were found in Sudan. Other species such as Egyptian Cobra (*Naja haje*) and the Black‐necked Spitting Cobra (*Naja nigricollis*) were also reported.^10^

Various presentations of bleeding after snakebite have been reported in the literature. Hemoperitoneum, intracranial bleeding, hematuria, hemoptysis, and ischemic strokes are examples.^11^ Up to our knowledge, hemothorax following snake bite has not been reported in a Sudanese patient. Similar cases of snake bite complicated by hemothorax were observed in a girl in India and a boy from San Francisco.^11^, ^12^

Snake toxins induced activation of the clotting pathway results in direct endothelial damage and subsequent coagulopathy.^13^ Despite this fact, the exact pathophysiology for developing of hemothorax after snake bite is not fully understood.^11^ In such condition, chest radiograph along with PT and INR is mandatory.

There is no local‐specific antivenom manufactured in Sudan, and thus, snake bite victims are treated by antivenoms imported from abroad.^14^ Usage of antivenom induces binding and neutralization of the toxins and thus stops further consumption.^15^ Although there is controversy surrounding the benefit of snake venom in the recovery of clotting function,^13^ we reported improving in our patient's clotting function after using antivenom. In contrast, the use of vitamin K and fresh‐frozen plasma will result in more rapid restoration of the clotting function and thus reduce the possibility of hemorrhage.^16^

## 4 CONCLUSIONS

Hematological complications are common after snake bite. The development of shortness of breath and percussive dullness with decreased air entry should raise the suspicion of hemothorax. Insertion of chest tube is necessary in the management of such cases.

## CONFLICTS OF INTEREST

The authors report no conflicts of interest in relation to the present study.

## AUTHOR CONTRIBUTIONS

MAD and EAR wrote the manuscript. EH revised the article.

## ETHICAL APPROVAL

The consent is available in the medical record and with the corresponding author.

## Data Availability

The data that support the findings of this study are available from the corresponding author upon reasonable request.
